# Caloric Restriction Decreases Orthostatic Tolerance Independently from 6° Head-Down Bedrest

**DOI:** 10.1371/journal.pone.0118812

**Published:** 2015-04-27

**Authors:** John P. Florian, Friedhelm J. Baisch, Martina Heer, James A. Pawelczyk

**Affiliations:** 1 Navy Experimental Diving Unit, Panama City, Florida, United States of America; 2 Department of Kinesiology, Noll Laboratory, The Pennsylvania State University, University Park, Pennsylvania, United States of America; 3 DLR-Institute of Aerospace Medicine, Cologne, Germany; University of Buenos Aires, Faculty of Medicine. Cardiovascular Pathophysiology Institute., ARGENTINA

## Abstract

Astronauts consume fewer calories during spaceflight and return to earth with an increased risk of orthostatic intolerance. Whether a caloric deficiency modifies orthostatic responses is not understood. Thus, we determined the effects of a hypocaloric diet (25% caloric restriction) during 6° head down bedrest (an analog of spaceflight) on autonomic neural control during lower body negative pressure (LBNP). Nine healthy young men completed a randomized crossover bedrest study, consisting of four (2 weeks each) interventions (normocaloric bedrest, normocaloric ambulatory, hypocaloric bedrest, hypocaloric ambulatory), each separated by 5 months. Muscle sympathetic nerve activity (MSNA) was recorded at baseline following normocaloric and hypocaloric interventions. Heart rate (HR) and arterial pressure were recorded before, during, and after 3 consecutive stages (7 min each) of LBNP (-15, -30, -45 mmHg). Caloric and posture effects during LBNP were compared using two-way ANOVA with repeated measures. There was a strong trend toward reduced basal MSNA following caloric restriction alone (normcaloric vs. hypocaloric: 22±3 vs. 14±4 burst/min, p = 0.06). Compared to the normocaloric ambulatory, both bedrest and caloric restriction were associated with lower systolic blood pressure during LBNP (p<0.01); however, HR responses were directionally opposite (i.e., increase with bedrest, decrease with caloric restriction). Survival analysis revealed a significant reduction in orthostatic tolerance following caloric restriction (normocaloric finishers: 12/16; hypocaloric finishers: 6/16; χ2, p = 0.03). Caloric restriction modifies autonomic responses to LBNP, which may decrease orthostatic tolerance after spaceflight.

## Introduction

Orthostatic intolerance (OI), the inability to maintain blood pressure (BP) while standing, affects over 500,000 individuals in the United States [1], and up to 64% of astronauts upon return from microgravity [2]. Symptoms include fatigue, headache, nausea, presyncope, and occasionally syncope, resulting from inadequate cerebral perfusion upon standing [1]. Though the etiology of OI has been extensively researched, its pathophysiology is still poorly understood. One well-known factor predisposing astronauts to OI is a reduction in blood volume [3], leading to decreased cardiac filling and diminished stroke volume [4]. Other potential factors contributing to the reduced cardiac filling and systemic blood distribution include cardiac atrophy, altered cardiovascular neurohumoral regulation, diminished carotid-cardiac baroreflex responsiveness [5], and augmented peripheral pooling (e.g. legs, abdomen) and venous compliance [6].

Despite the effort to simulate the effects of spaceflight employing bedrest, water immersion, and other interventions, the role of diet has been largely unexplored. Astronauts consume ~25% fewer calories during spaceflight than necessary to maintain body weight [7,8]. Inadequate food intake can severely impact endocrine, muscular, and cardiovascular performance. Even moderate caloric restriction (CR) can impact fluid homeostasis resulting in reduced blood volume and cardiovascular function [9]. For example, pilots who fasted had reduced weight (-2.7%), plasma volume (-7%), augmented HR, and diminished pulse pressure during orthostasis when compared to responses before fasting [10]. Furthermore, CR reduces heart rate (HR), BP, and norepinephrine turnover in rats [11,12] and in obese normotensive humans following a reduction in weight [13]. However, it is not known how actual or simulated microgravity in conjunction with hypocaloric intake affect the cardiovascular and sympathetic neural responses to orthostasis in healthy individuals.

Therefore, the objective of the current study was to determine the influence of hypocaloric intake coupled with ambulation or bedrest on hemodynamic variables and muscle sympathetic nerve activity (MSNA) during orthostatic stress. We hypothesized that chronic hypocaloric energy supply would intensify the development of OI.

## Materials and Methods

### Ethics Statement

Volunteers gave their written informed consent to participate in the study, which conformed to the latest revision of the Declaration of Helsinki and was approved by the Ethical Committee of the ‘Arztekammer Nordrhein’, Dusseldorf, Germany.

### Subjects

Nine healthy active men (age: 23.8 ± 3.0 years; BMI: 22.8 ± 3.2 kg/m^2^) who were not endurance trained [14] completed a randomized crossover bedrest and CR study to simulate the effects of spaceflight. Subjects were enrolled if they met all of the following inclusion criteria: physical examination, ECG, urinalysis and routine laboratory without clinically relevant findings, total cholesterol ≤5.2 mmol l^-1^, LDL ≤3.4 mmol l^-1^, HDL ≥0.9 mmol l^-1^, and fasting glucose ≤5.9 mmol l^-1^. Exclusion criteria included hyperlipidemia, arterial hypertension, diabetes, regular medication and/or treatment with drugs within the last 6 weeks, acute or chronic illness, smoking within a period of 1-year preceding the study, and drug and/or alcohol abuse.

### Study Design

The study was performed in a randomized cross-over design as part of a multi-disciplinary project evaluating the effects of simulated microgravity and hypocaloric nutrition on cardiovascular and sympathetic nervous function. Thus, information from several of the supporting references was derived from the current study design. The subjects participated in 4 study phases that were separated by at least 5 months to allow complete recovery of the participants. Each study phase started with a 9-day adaptation period followed by a 14-day intervention period; in each of the 4 intervention periods the participants were exposed to either bedrest or ambulatory control conditions, while receiving either a tailored normocaloric or hypocaloric diet. Cardiovascular and sympathetic responses to lower body negative pressure (LBNP) were investigated on day 14 of the intervention period. All four study phases were identical with respect to environmental conditions and study protocol; only the variables posture (bedrest or ambulation) and energy intake (normocaloric or hypocaloric) were changed.

### Ambulatory and Bedrest Conditions

The participants resided in a metabolic ward (Institute of Aerospace Medicine, German Aerospace Center, Cologne, Germany) during the entire period of the four interventions. Room temperature (24°C) and relative humidity (50%) were kept constant in the metabolic ward and the laboratory. During the bedrest phases, all activities, including food intake, using the toilet, showering, and weighing, were carried out in the 6° head-down-tilt or horizontal position. 6° head-down-tilt was chosen because it is a validated model for simulation of microgravity [15]. Though the induced cardiovascular changes occur more rapidly, their nature and extent is very similar to those observed in supine position. During the ambulatory control phases, the participants maintained upright position during the day and were allowed to walk around in the ward. Though they were not allowed to exercise voluntarily, they followed a light exercise protocol (including bicycle ergometry ~125 W—15 min twice/day).

### Diet

During all adaptation and recovery periods as well as during the normocaloric, ambulatory intervention the participants received a normocaloric standard diet. Energy requirements were calculated for each individual according to the FAO/WHO equations [16]: participants received a specifically prepared diet containing 1.4 times their basal metabolic rate (BMR). Ten percent of the total calories was added to account for dietary-induced thermogenesis. The average caloric intake was 11.4±1.3 MJ/day, which consisted of 1 g protein per kg body weight/day, 50 ml water per kg body weight/day, 2.5 mmol sodium per kg body weight/day, 1000 mg calcium/day, and vitamin D 400 IU/day administered as fixed dose tablets. Dietary protein, fat (saturated and polyunsaturated fatty acids), and carbohydrate intakes were calculated according to dietary reference intake values [17] (i.e. 1.0 g /kg body mass /day as protein, 30% as fat, and the remaining part as carbohydrates). The German recommended dietary intake levels were used for nutrients without experiment-specific requirements. No caffeine, methylxanthine, or alcohol was allowed. Six meals were prepared daily which included three main meals and three snacks. The volunteers received and ate the exact amount of food that was predefined in their individual menu. During the intervention periods, the average energy content of the diet was modified as follows: normocaloric ambulatory: 11.4±1.3 MJ/day; hypocaloric ambulatory: about 25% decrease in calories compared to standard diet, 9.0±1.1 MJ/day; normocaloric bedrest: energy content was reduced from the standard diet to adjust to the reduced physical workload; participants received a diet containing 1.1 (instead of 1.4) times their BMR, 9.4±1.4 MJ/day; hypocaloric bedrest: about 25% decrease in calories compared to the respective normocaloric bedrest phase, 7.7±0.9 MJ/day. Reduction in energy intake was mainly achieved by reduction of fat intake to a minimum level of 60g/day in order to keep the recommended level of essential fatty acids. Protein intake was kept constant during all study phases. Other than fat, nutrient composition of each experiment day was identical to the normocaloric study periods.

### Heart Rate and Arterial Pressure

Heart rate was derived from a surface electrocardiogram. Beat-to-beat finger arterial pressure was measured by finger photoplethysmography (Portapres, Amsterdam, The Netherlands), and auscultatory BP was taken at baseline and at the end of each stage during the protocol.

### Muscle Sympathetic Nerve Activity

Peroneal nerve muscle sympathetic activity (MSNA) was recorded as described previously [18]. Briefly, the nerve was located with cutaneous electrical stimulation (Isostim A320, World Precision Instruments). A tungsten reference electrode (FHC, Bowdoinham, ME, USA) was inserted subcutaneously, ~2 cm from the nerve, and a tungsten recording electrode with an uninsulated tip diameter of ~10 μm was inserted through the skin near the nerve. Adjustments of the recording electrode position were made according to auditory signals generated by impaled nerves. Both electrodes were connected in series to a differential preamplifier and an amplifier (NASA, Houston, TX, USA), isolated by two 100 mA current limiters. The nerve signal was amplified (total gain 40,000 to 80,000), band-pass filtered (high pass of. 7 kHz and low pass of 2–3 kHz), and then full-wave rectified and smoothed with a resistance-capacitance circuit (time constant, 0.1 s) to produce a recording of “integrated” MSNA. Satisfactory recordings of MSNA were defined by pulse-synchronous bursts that increased during end-expiratory apnea or Valsalva straining and did not change during tactile or auditory stimulation.

### Lower Body Negative Pressure

Suction was applied with subjects supine in lower body chambers sealed at the iliac crests. The chamber, developed by the Deutsche Agentur Raumfahrtagelegenheiten, was made of collapsible fabric, and had windows to allow leg access for microneurography. After 7 minutes of baseline recording, steady pressure was applied at -15, -30, and -45 mmHg, in fixed order, for 7 minutes each, or until presyncope. Data were analyzed during the third to fifth minute of each 7 minute period, and blood was drawn at the end of each segment. Presyncope was defined as a decrease in systolic BP (SBP) to <80 mmHg; a decrease in SBP to <90 mmHg associated with symptoms of lightheadedness, nausea, or diaphoresis; or progressive symptoms of presyncope accompanied by a request from the subject to discontinue the test. Since the LBNP protocol consists of a set level of negative pressure and time at each stage, not all subjects were expected to reach an endpoint of presyncope.

### Data Analysis

Three minutes of data from recordings of MSNA, BP, and HR collected at baseline were each averaged to a single value. Likewise, BP and HR data for minutes 2–5 of each stage of LBNP were averaged to a single point. Data where subjects became presyncopal are not included in the analysis.

Student’s paired t-test was utilized to determine differences between resting MSNA values. A repeated measures analysis of variance (ANOVA) was conducted to determine the influence of caloric intake, posture, and time on MSNA and hemodynamic variables. Least squares means with Bonferroni correction were performed when appropriate to detect where differences between factors occurred. The level of significance was set at α = 0.05. Values are presented as means ± SEM, unless indicated otherwise.

## Results

### Subject characteristics

**Fig 1:**
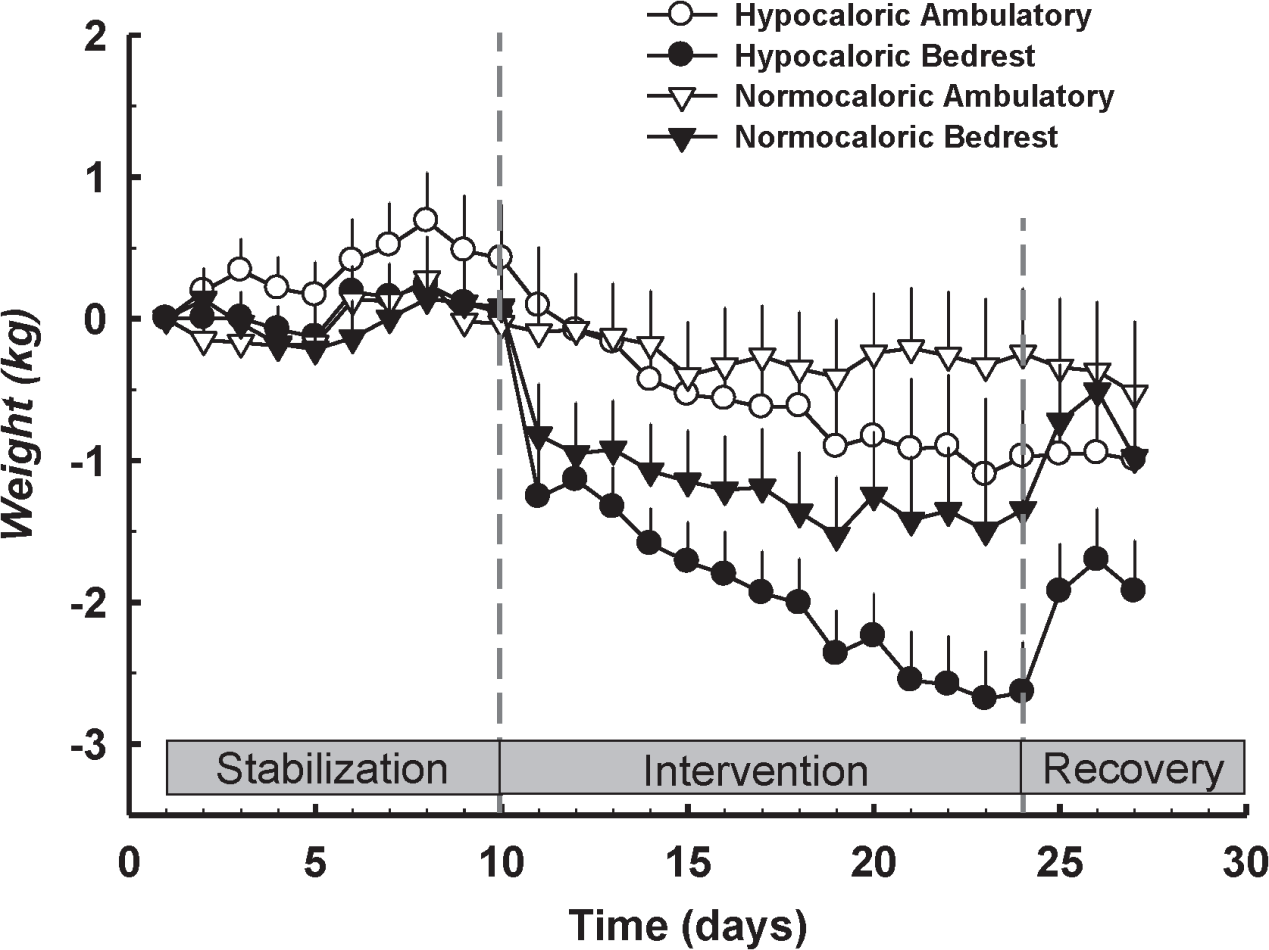
Change in subject weight before, during, and after each intervention. Data are presented as mean ± SEM. Weight was significantly reduced vs. baseline and control following all experimental interventions.

The subject clinical characteristics at screening are presented in Table 1. All subjects were young, healthy, normotensive, and nonobese. Subject weights were not different at baseline for each of the interventions: hypocaloric ambulatory (79.8±3.6 kg), hypocaloric bedrest (78.7±3.3 kg), normocaloric ambulatory (76.9±3.2 kg), normocaloric bedrest (78.1±2.8 kg). Fig 1 depicts the change in subject weight over the course of each intervention. Weight significantly decreased from baseline for all interventions except for control (normocaloric ambulatory).

**Table 1:** Subject characteristics.

Subjects	N = 9
Age (years)	24±3 (21–29)
Height (cm)	182±6
Weight (kg)	76±7
BMI (kg m^-2^)	23±9
Total cholesterol (mmol l^-1^)	4.2±0.6
HDL (mmol l^-1^)	1.3±0.3
LDL (mmol l^-1^)	2.5±0.3
SBP (mmHg)	123±6
DBP (mmHg)	78±8

Values are mean ± SD. BMI, body mass index; HDL, high density lipoprotein; LDL, low density lipoprotein; SBP, systolic blood pressure; DBP, diastolic blood pressure.

### Heart Rate and Arterial Pressure

**Fig 2:**
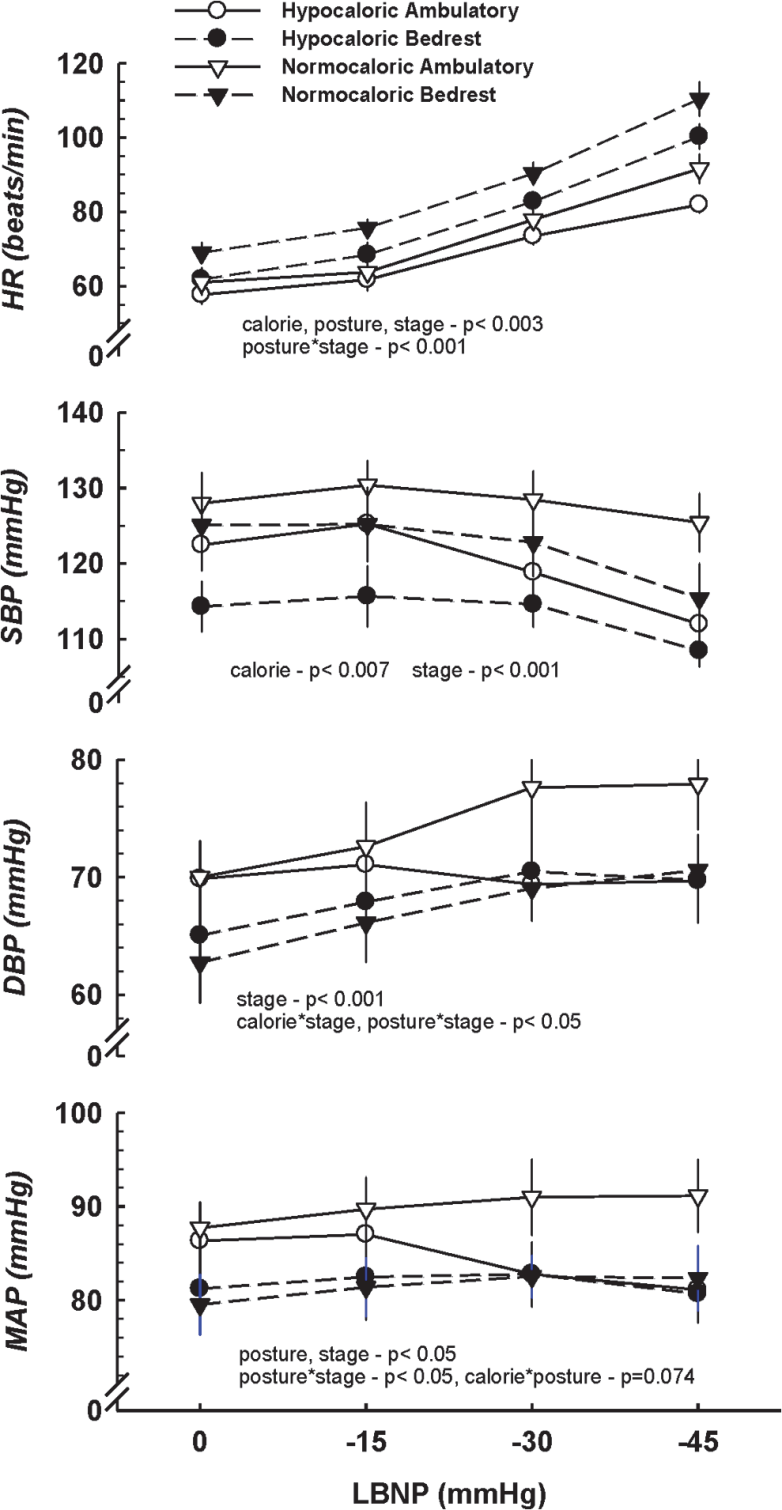
Systemic hemodynamic responses to 3 stages of LBNP. Data are presented as mean ± SEM. The main effects calorie, posture, and stage are indicated below each respective graph. Following bedrest, HR at rest and during LBNP is augmented (posture * time interaction); however, tachycardia was attenuated with CR. Both CR and bedrest are associated with lower SBP during LBNP. MAP tended to decrease (p = 0.074) during LBNP following CR alone.

Hemodynamic measurements before and during lower body suction following each intervention are depicted in Fig 2. Heart rate was significantly lower at baseline and throughout LBNP following both CR trials, whereas bedrest, independent of caloric intake, was associated with a higher HR. As expected, HR increased during the 3 stages of LBNP (p<0.001), and the cardioacceleration was greater following bedrest, independent of caloric intake (p<0.001). Caloric restriction was associated with a lower SBP during LBNP (p = 0.007), and SBP decreased in all interventions by the last stage of LBNP (p<0.001). No main effects for calorie or posture were identified for diastolic BP (DBP); however, CR abolished, and bedrest enhanced, the increase in DBP during LBNP. A trend toward reduced mean arterial pressure (MAP) following CR alone was present during LBNP (p = 0.07).

### Muscle Sympathetic Nerve Activity

**Fig 3:**
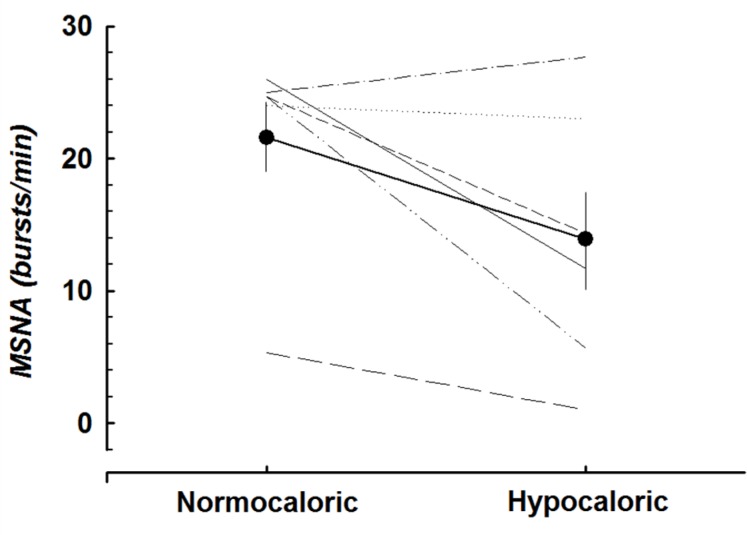
MSNA after caloric restriction. Baseline MSNA values before and after the hypocaloric ambulatory trial for 6 subjects. There was a strong trend toward reduced basal MSNA following CR (p = 0.06).

Microneurography was not attempted during the 1^st^ intervention; therefore, only MSNA data for the hypocaloric and normocaloric ambulatory interventions were examined. Additionally, due to the large attrition of subjects and microneurography electrode shifts with increasing lower body suction, only baseline MSNA data for six subjects before and after the hypocaloric ambulatory intervention could be analyzed, showing a strong trend toward reduced basal MSNA (p = 0.06) following CR (Fig 3).

### Tolerance to Lower Body Suction

**Fig 4:**
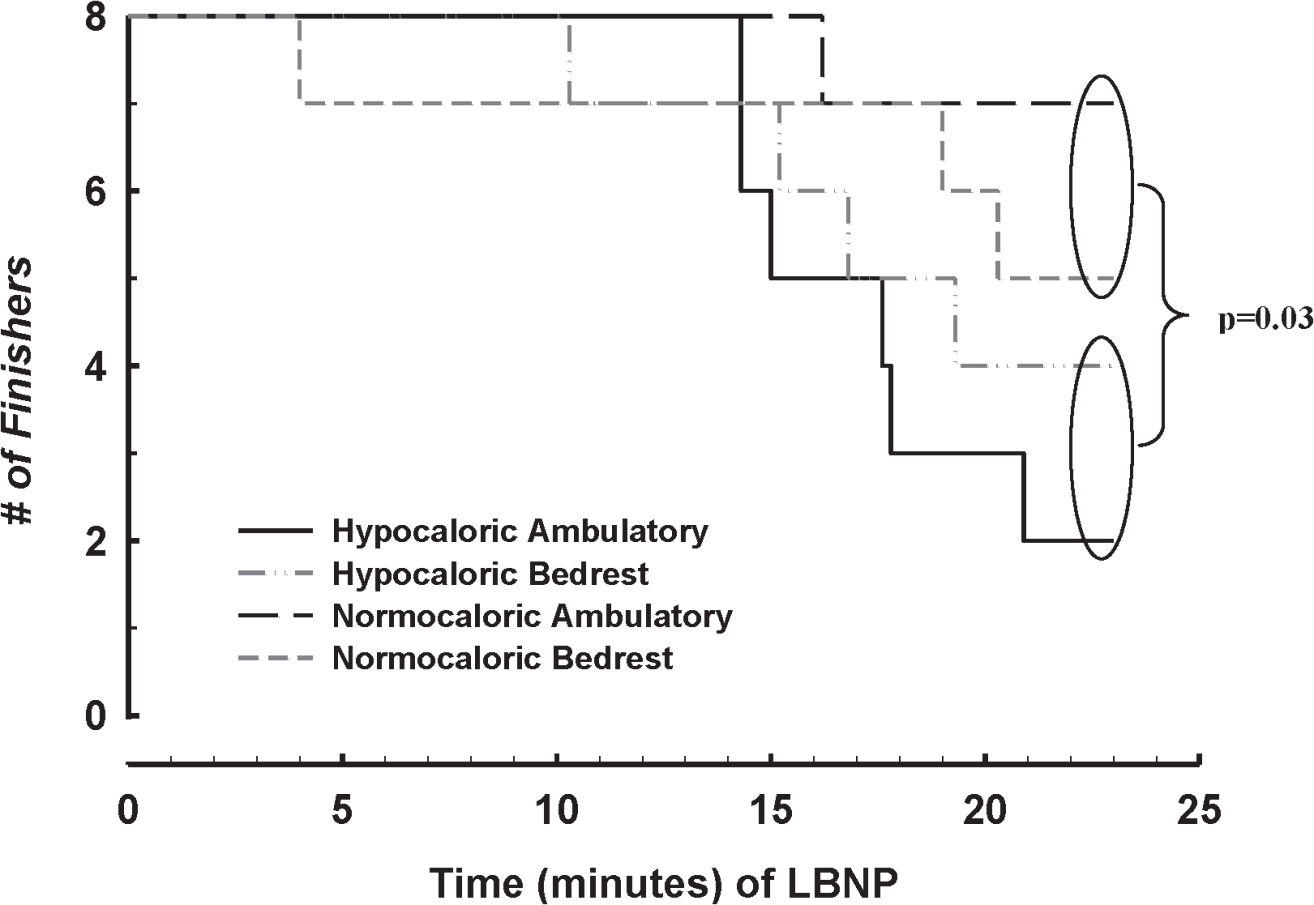
LBNP survival analysis. Survival analysis comparing the number of finishers and time of failure for non-finishers during LBNP. Caloric restriction vs. normocaloric intake reduces orthostatic tolerance (χ^2^, p = 0.03), and CR alone shows a tendency (χ^2^, p = 0.09) toward reduced orthostatic tolerance independent of bedrest.

Data for 8 of the 9 subjects were analyzed for LBNP due to technical problems with the data acquisition system. The number of individuals remaining at a given time of LBNP is presented in Fig 4. Seven of 8 individuals completed the entire LBNP protocol following the normocaloric ambulatory intervention, and half the subjects completed the LBNP protocol following both bedrest interventions. Interestingly, only 2 of 8 subjects finished after the hypocaloric intervention alone. Survival analysis indicates a significant effect (p = 0.03) of CR compared to normocaloric intake to reduce orthostatic tolerance and a tendency (p = 0.09) toward reduced orthostatic tolerance with CR independent of bedrest when compared with the other interventions. Moreover, CR alone reduced the cumulative stress index (CSI; Σ(LBNP * time) compared to the pre-intervention CSI (587±32 vs. 483±47 mmHg * min; p = 0.04).

## Discussion

This investigation was conducted to determine the effects of hypocaloric low-fat intake, bedrest, and their combination on neural and cardiovascular control in healthy young volunteers at rest and during orthostatic stress.

### Effect of Caloric Restriction

The major findings of the present study are as follows: (1) CR decreased basal sympathetic outflow in lean, normotensive subjects; (2) tolerance to lower body suction was reduced following 14 days of a hypocaloric, low-fat diet alone; (3) HR and SBP at baseline and throughout LBNP were significantly attenuated following hypocaloric interventions compared with normocaloric interventions, and (4) CR with ambulation dramatically affected the BP responses to LBNP at -30 and -45 mmHg, thus contributing to the reduced orthostatic tolerance.

Previous studies have reported reductions in hemodynamic variables following CR in animals and humans. Similar to this investigation, 2 weeks of 60% of normal caloric intake decreased MAP and HR in normotensive, nonobese mice under thermoneutral conditions [11], and 2 weeks of low-fat consumption reduced SBP/DBP by 7/3 mmHg and HR measured over 24 hours [19,20]. To our knowledge no other studies have examined the effects of reduced caloric/fat intake on hemodynamic responses to LBNP. However, our results are supported by an investigation that assessed BP and HR responses to 10-min of 80° head-up tilt before and after 1 month of fasting in healthy male pilots [10]. In concord with our study, fasting reduced SBP at baseline and during tilt; however, cardioacceleration was enhanced during tilt, in opposition to the attenuated HR response observed in the present investigation.

The reduction in HR following CR from fat is most likely attributable to both a reduction in cardiac sympathetic activation and increased parasympathetic inhibition. For example, seven days of 50% CR increased the interbeat interval in mice [21]. Additionally, spectral analysis points toward cardiac sympathetic activation with lipid infusion [22,23], and a reversal in sympathetic/parasympathetic balance following a reduction in plasma fatty acids [22]. Reduced sympathetic outflow has been reported in previous investigations following caloric/fat restriction in animals and overweight or obese individuals [12,13,24,25,26]; however, to our knowledge, this is the first study to show diminished sympathetic outflow in lean, normotensive humans.

### Why Does Caloric Restriction Reduce Blood Pressure During LBNP?

Several mechanisms may have contributed to the reduced cardiovascular responsiveness and orthostatic tolerance with caloric/fat restriction including the following: (1) reduced blood volume, (2) modulation of autonomic function, (3) altered baroreflex function, and (4) altered vascular smooth muscle tone and responsiveness.

Consistent with the loss of weight (and gain during recovery) in this study, short-term CR produces a 7–8% fall in plasma volume in lean [10] and obese individuals [27]. Thus, reduced caloric intake may contribute to the 10–15% fall in blood volume during spaceflight [6]. In fact, it has been shown that nutritional energy supply during missions is associated with chronic total body mass (presumably fluid) loss or gain during spaceflight [28]. Therefore, the reduced orthostatic tolerance observed in this investigation may be explained, at least in part, by reduced blood volume [29].

The cause of the reduced blood volume is not entirely clear as there is a paucity of available information on the mechanisms of how chronic hypocaloric nutrition influences fluid balance and renal regulation. Sodium intake was consistent between phases and throughout the intervention. Recent terrestrial [30] and spaceflight [28,31] studies suggest that instead of altered renal function and sodium handling, the hypocaloric nutrition and activation of alternative energy resources may have altered the water binding capacity and thus the distribution and cumulative balance of total body water. Specifically, fluid may have shifted from the vascular bed into the extravascular space together with slow total fluid loss over the course of the hypocaloric ambulatory intervention. The changes during the hypocaloric bedrest intervention likely resulted from a combination of the compartmental fluid shifts that occurred from CR alone together with the diuresis from bedrest [29].

Caloric/fat restriction and fasting suppress sympathetic activity in animals [12,26] and overweight or obese individuals [13,24,25]. Diminished sympathetic outflow may be mediated by a reduction in leptin and/or insulin levels, which are altered acutely by caloric intake independent of changes in body weight [32]. Leptin has also been shown to be reduced following caloric restriction and hindlimb unloading in the rat model (Baek *et al*., 2008). Additionally, free fatty acids modulate leptin [33,34,35,36] and insulin [23,37,38], and directly activate the sympathetic nervous system [39,40,41]. Thus, a reduction in free fatty acids (as may occur in moderate but not severe CR) may directly or indirectly reduce central sympathetic outflow. The reduction in sympathetic activity at rest in the present study supports this idea. Moreover, it is possible that the sympathetic response during LBNP is also diminished [19], contributing to the decreased BP and LBNP tolerance.

Though carotid-cardiac baroreflex responsiveness may be reduced after spaceflight [5], previous studies have suggested that CR alone enhances baroreflex function [13,42]. Therefore, we would have expected a greater tachycardic response since BP decreased during LBNP. Interestingly, a blunted HR response was observed following CR in this study.

Reduced fat intake and plasma fatty acids may decrease vascular tone through a variety of mechanisms including increased insulin sensitivity [43], nitric oxide synthase and nitric oxide production [44], enhanced endothelium-dependent and—independent vasodilation as shown by collaborators in STBR-IP [45], and reduced α-adrenergic sensitivity [46]. Additionally, CR reduces oxidative stress [45] which has been linked to hypertension [47].

### Effect of Bedrest

Consistent with other studies of simulated or actual microgravity, half the participants in this experiment were unable to finish the protocol during orthostatic stress. We cannot determine the exact underlying mechanism(s) associated with the altered hemodynamic response and reduced orthostatic tolerance from our results. Moreover, MSNA could not be recorded due to experimental limitations for the normocaloric bedrest intervention. However, our findings are in accord with a bedrest study of similar duration [29]. Following 18 days of 6° head-down bedrest, subjects had reduced LBNP tolerance through a combination of cardiac atrophy [48] and hypovolemia, coupled with augmented sympathetic activation [29]. The elevated basal HR and enhanced cardioacceleration during LBNP following bedrest alone in the present study may be indicative of a hypovolemic, hyperadrenergic form of orthostatic intolerance [29] instead of a form resulting from downregulated baroreflex control [5].

### Combined Effect of Bedrest and Caloric Restriction

When hypocaloric intake was combined with bedrest, tolerance to lower body suction mirrored the response observed during bedrest alone. Thus, our hypothesis that CR would intensify the development of OI during bedrest is not supported. Diastolic BP at rest and during LBNP was also similar to bedrest alone, whereas HR was in between that of the normocaloric bedrest and hypocaloric ambulatory interventions, and SBP was lower throughout. These data may, therefore, indicate that caloric/fat restriction may antagonize the enhanced sympathetic activation and endothelial dysfunction [45] of bedrest and/or enhance the hypovolemia resulting from bedrest or CR alone. The lower weight associated with the hypocaloric bedrest trial supports this notion.

### Limitations

Several factors limit our interpretation of the results. First, the study was relatively underpowered; though ten subjects were recruited, only nine completed the study. Further, data for one subject were not analyzed due to technical problems with data acquisition during one of the trials. Consequently, strong trends were identified for certain variables (e.g. MAP) following the hypocaloric ambulatory intervention alone which may have achieved significance with data from more subjects. Nevertheless, the noted changes, whether as trends or significant changes, highlight important findings that merit further study. Second, microneurography was not attempted during the 1^st^ phase, limiting our analysis of MSNA data for all phases. Third, blood volume and cardiac output were not measured; therefore, we can only speculate the contribution of these factors from other related studies. Finally, the exact neurohumoral and renal mechanisms underlying the physiological responses to CR and bedrest cannot be ascertained from the current investigation.

### Conclusion

This study provides the first evidence that 14 days of caloric/fat restriction in healthy men decreases resting MSNA and LBNP tolerance. The predisposition to orthostatic intolerance may result from blunted neurogenic and cardiovascular responses to orthostatic stress. Hypocaloric intake combined with bedrest may offset the adaptation to simulated microgravity alone. Further acute and chronic mechanistic studies are required to examine possible neural and hormonal mediators of the cardiovascular responses to CR alone or CR combined with bedrest.
